# Degradation of collagen I by activated C1s in periodontal Ehlers-Danlos Syndrome

**DOI:** 10.3389/fimmu.2023.1157421

**Published:** 2023-03-07

**Authors:** Albert Amberger, Johanna Pertoll, Pia Traunfellner, Ines Kapferer-Seebacher, Heribert Stoiber, Lars Klimaschewski, Nicole Thielens, Christine Gaboriaud, Johannes Zschocke

**Affiliations:** ^1^ Institute of Human Genetics, Med. Univ. Innsbruck, Innsbruck, Austria; ^2^ Department of Conservative Dentistry and Periodontology, Med. Univ. Innsbruck, Innsbruck, Austria; ^3^ Institute of Virology, Med. Univ. Innsbruck, Innsbruck, Austria; ^4^ Institute of Neuroanatomie, Med. Univ. Innsbruck., Innsbruck, Austria; ^5^ Univ. Grenoble Alpes, Commissariat à l’énergie atomique et aux énergies alternatives (CEA), Centre National de la Recherche Scientifique (CNRS), Institut de Biologie Structurale (IBS), Grenoble, France

**Keywords:** Ehlers-Danlos Syndrome, collagen I, periodontites, complement activation, C1r protease

## Abstract

Periodontal Ehlers-Danlos syndrome (pEDS) is an autosomal dominant disorder characterized by early-onset periodontitis leading to premature loss of teeth, lack of attached gingiva and thin and fragile gums leading to gingival recession. Connective tissue abnormalities of pEDS typically include easy bruising, pretibial plaques, distal joint hypermobility, hoarse voice, and less commonly manifestations such as organ or vessel rupture. pEDS is caused by heterozygous missense mutations in *C1R* and *C1S* genes of the classical complement C1 complex. Previously we showed that pEDS pathogenic variants trigger intracellular activation of C1r and/or C1s, leading to extracellular presence of activated C1s. However, the molecular link relating activated C1r and C1s proteases to the dysregulated connective tissue homeostasis in pEDS is unknown. Using cell- and molecular-biological assays, we identified activated C1s (aC1s) as an enzyme which degrades collagen I in cell culture and in *in vitro* assays. Matrix collagen turnover in cell culture was assessed using labelled hybridizing peptides, which revealed fast and comprehensive collagen protein remodeling in patient fibroblasts. Furthermore, collagen I was completely degraded by aC1s when assays were performed at 40°C, indicating that even moderate elevated temperature has a tremendous impact on collagen I integrity. This high turnover is expected to interfere with the formation of a stable ECM and result in tissues with loose compaction a hallmark of the EDS phenotype. Our results indicate that pathogenesis in pEDS is not solely mediated by activation of the complement cascade but by inadequate C1s-mediated degradation of matrix proteins, confirming pEDS as a primary connective tissue disorder.

## Introduction

Ehlers Danlos Syndromes (EDS) are a heterogeneous group of monogenic connective tissue disorders with variable clinical manifestations including joint hypermobility and dislocation, skin hyperelasticity and fragility with abnormal scar formation, and other features such as aortic dissection, blood vessel fragility, organ rupture, and often chronic pain (1, 2). One of the rare types of EDS is periodontal Ehlers-Danlos Syndrome (pEDS) (OMIM 130080 and 617174, prevalence unknown), an autosomal dominant disorder characterized by lack of attached gingiva, early severe periodontitis causing premature loss of teeth, and variable other manifestations such as mild distal hypermobility, skin fragility, easy bruising and pretibial hyperpigmentation, recurrent infections, and often asymptomatic leukoencephalopathy (3). pEDS is caused by heterozygous missense or in-frame insertion/deletion mutations in the *C1R* or *C1S* genes (4). These genes code for the homologous serine proteases C1r and C1s of the C1 complement complex and are known for their regulatory function in initiating the classical complement pathway (5). Pathogenesis of pEDS thus differs from the other EDS types, which are usually due to a primary disturbance of extracellular matrix proteins, mostly involving collagens or collagen-modifying enzymes. The molecular mechanism linking C1r and C1s to dysregulated connective tissue homeostasis in pEDS has so far been undetermined.

C1r and C1s are modular proteins with the same distinct domain structure: two CUB domains, separated by an EGF-like domain, two CCP domains and a serine protease domain. The CUB domain (C1r/C1s, Uegf and BMP-1) is a structural motif of about 110 amino acids and together with the EGF-like domain mediates the C1r2C1s2 hetero-tetramer formation (6). Additionally, the C1r CUB1 and CUB2 domains and the C1s CUB1 domain interact with conserved lysine residues in the collagen-like stems of C1q in the assembly of the C1 complex (7). The C1r2C1s2 hetero-tetramer thus provides six collagen interaction sites – one for each stem of the C1q hexamer (8). The C1 complex interacts with the Fc domain of IgG or IgM antibodies bound to microorganisms or infected cells. Upon binding, a series of proteolytic events is initiated, which starts by auto-activation of C1r and subsequently activation of C1s. This initiates a cascade of events leading to the formation of the anaphylatoxins C3a and C5a and the lysis of microbial targets *via* the multimeric membrane-attack-complex (C5b-9) (5, 9).

We recently showed that pEDS pathogenic variants cause uncontrolled activation of the C1r and/or C1s serine proteases, leading to extracellular presence of activated C1s (aC1s). In fibroblasts from pEDS patients, variant C1r is auto-activated intracellularly and subsequently activates C1s even in the presence of C1 inhibitor (10). Similarly, pEDS pathogenic *C1S* variants expose a normally inaccessible cleavage site that escapes normal physiological control and triggers abnormal C1s activation (11). Pathological production of aC1s thus seems to be the common pathogenic element in pEDS associated with both, *C1R* and *C1S* variants.

A number of studies have suggested a variety of biological functions of C1s beyond its role in complement activation (12). In 1990, Yamaguchi and colleagues reported that aC1s may proteolytically cleave collagens I and II in an *in vitro* assay (13). In addition, C1s has also been reported to degrade the matrix proteoglycan decorin (14) and to activate matrix metalloproteinase 9 (15). However, these observations were never brought in context relevant for human disorders. In the present study, we examined a possible link between C1s activation and the extracellular collagen I network and turnover in pEDS, as well as potential therapeutic implications.

## Material and methods

### Cell culture

Primary skin fibroblasts were obtained with informed written consent from different pEDS patients and healthy controls (10) (patients cells were heterozygous for following variants c.149-150TC>AT; c.277G>T; c.902G>C, and c.926G>T, respectively). All fibroblasts were cultivated at 37°C and 5% CO_2_ in 75 cm^2^ cell culture flasks in 10 ml complete medium (500 ml Dulbecco’s Modified Eagle’s Medium - high glucose [DMEM, Sigma Aldrich/Merck, Darmstadt, Germany, D6546], 50 ml heat-inactivated fetal bovine serum [FBS, Gibco/Fisher Scientific, Vienna, Austria, 10500-064], 5ml Penicillin/Streptomycin [Sigma Aldrich, P0781]). All cell lines were tested every 4 to 6 weeks for mycoplasma infection using a PCR detection kit (Minerva Biolabs, Berlin, Germany) according to the manufacturer’s instructions.

### SDS-PAGE and immunoblotting

Western blot analyses were performed under denaturing and reducing conditions (unless indicated otherwise) using standard procedures. Whole cell lysates (20 µg) and concentrated cell culture supernatants were generated using RIPA buffer containing HALT™ protease inhibitor cocktail (1:100) and were separated in a 10% SDS gel (Biorad Laboratories, Vienna, Austria, #4561031). Page Ruler pre-stained protein ladder (Fisher Scientific, #26616) was used for determining protein molecular weight. Proteins were blotted onto a PVDF membrane *via* wet blotting (1 h, 100 V, 4°C) and blocked in blocking buffer (0.05% Tween 20, 5% milk (w/v) in PBS) at room temperature for one hour. Membranes were probed with C1s specific antibodies (Abcam, Cambridge, UK, ab185212) diluted 1:2000 in blocking buffer. Collagen I was detected using specific anti-collagen I (Abcam, ab138492; 1:1000). HRP-conjugated secondary antibody (Dako, Glostrup, Denmark, P0448) was used 1:10000 in blocking buffer. Alpha tubulin was used as loading control for PAGE under reducing conditions. Signals were detected with ECL (GE Healthcare, Vienna, Austria, RPN3243) and developed on x-ray films (Amersham Hyperfilm ECL, Distributor: VWR, Vienna, Austria).

### Immunofluorescence

Cover slips (Paul Marienfeld, Lauda-Königshofen, Germany, #011520) were washed with ethanol 70%, placed in a 24 well plate and air dried under the cell culture hood at RT for 30 min. Fibroblasts (1.5 x 10^5^) were seeded and cultivated in 1 ml of DMEM medium at 37°C for 24 h. After 24 h cells were fixed with 4% paraformaldehyde (Merck, #100496) solution at RT for 10 min and then washed three times for 5 min with 200 μl of a 50 nM NH_4_Cl (Merck, 101145) solution to remove PFA remains. Finally, cover slips were washed with PBS 3 times for 5 min at RT and stored at 4°C. Fixed cells were blocked in 200 μl of 3% BSA (in PBS w/v) solution at RT for 30 min and then incubated with anti-collagen I antibody (Abcam, ab138492; 1:500). Thereafter, cells were washed three times with PBS for 5 min and incubated with Alexa Fluor-conjugated secondary antibodies (Alexa Fluor Plus 594, Invitrogen, A32740, 1:1000). Thereafter samples were stained with 200 μl DAPI solution (Sigma, D9542) at RT for 10 min. Subsequently, the cells were washed with PBS and embedded in Prolong™ Diamond solution (Invitrogen, P36961). Parallel cultures were fixed as described above and incubated with F-CHP (5 µM in PBS; 150 µl per slide) at 4°C over night. F-CHP was prepared as described in the protocol of the supplier (3Helix, Salt Lake City, UT, USA) (16). Thereafter, cells were washed and mounted in Prolong™ Diamond solution and subjected to confocal laser scanning microscopy.

### Confocal laser scanning microscopy

Specimens were mounted on stage of confocal microscope TCS SP8 (Leica Microsystems, Vienna, Austria) and images were acquired with a 63× glycerol (N.A. 1.3) objective in a stack of 1 μm thickness (z-interval of 170 nm). In total, five images from every coverslip were taken randomly. DAPI staining of the nucleus was performed to facilitate orientation in individual cells. Every experiment included different patient fibroblast lines and control fibroblast lines. Additionally, in every experiment negative controls without primary antibody were included. The images were deconvoluted using the standard “Deconvolution Express” algorithm in Huygens Software (version 18.10; SVI, Hilversum, Netherlands). For image processing, applications like channel selection and color changing were used. This step allows the separate presentation of the fluorescently labelled structures. We decided to represent the stained cell structures based on their emission color (Collagen I in red, CHP binding in green and DAPI in blue). After acquisition, images were saved as Leica LIF files and exported as Tiff data.

### Knock-down of C1s

Fibroblasts were seeded into culture flasks and grown for 24 h. Thereafter, cells were washed and transfected using commercial available siRNA (Integrated DNA Technologies, Leuven, Belgium) and siRNA transfection reagent (X-treme GENE siRNA transfection reagent; Roche, Vienna, Austria. #04476093001). Transfection was repeated after 48h and cultivated for an additional 48h. Then cell extracts were prepared and stored until further analysis.

### Purification of C1s and proteolytic assay

C1s was purified from human serum as described previously (17). Briefly, human serum was incubated with insoluble ovalbumin-anti-ovalbumin immune complexes at 4°C and the suspension centrifuged and washed in a calcium containing buffer. At this stage, activation of the C1 complex is only partial and complete activation is achieved by incubation of the suspension for 35 min at 30°C. Subsequent treatment with EDTA disrupts the bound C1 complex and selectively releases C1r and C1s that are further purified by anion-exchange chromatography. SDS PAGE analysis of purified C1s under reducing conditions confirms its complete activation state, as assessed by the presence of the A and B chains of the activated protease and by the absence of any proenzyme species. Twelve µg of collagen I were incubated with 2.5 µg activated C1s at 37°C over night in 20 µl incubation buffer (100 mM Tris-HCl (pH 7.4), 100 mM NaCl and 5 mM CaCl_2_). After the incubation samples were separated on 10% SDS-PAGE under reducing conditions and signals were visualized using coomassie staining solution (Brilliant blue R250, Sigma). Denaturation of collagen I was performed by heating the samples to 87°C for 10 min, immediately cooling at 4°C for additional 10 min followed by over night incubation with aC1s at 37°C. In some experiments the proteolytic C1s assay was performed at 33°C, 37°C, and 40°C, respectively. To examine the effect of the serine protease inhibitor PMSF on the proteolytic activity of aC1s some experiments were done in the presence of different concentrations of PMSF as indicated in the figure legends.

## Results

### Collagen I is degraded in fibroblasts from individuals with pEDS

**Figure 1:**
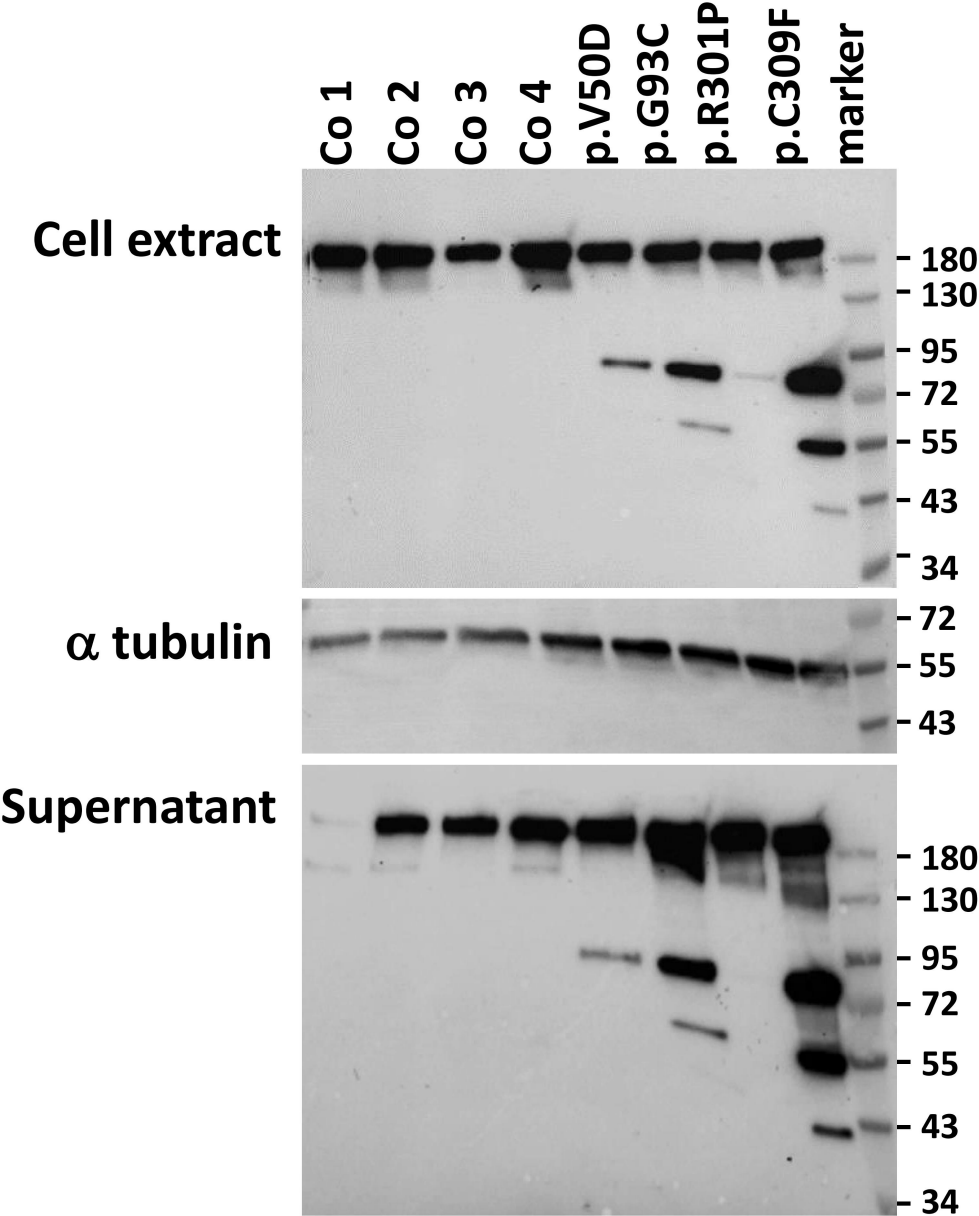
Western blot analysis of collagen I expression in control (Co) and pEDS fibroblasts. Cell extracts and cell supernatants were probed with an anti-collagen I antibody. The signal at approx. 150 kDa represents procollagen, which was present in all fibroblasts; absence of this signal in the supernatant of control 1 may be due to rapid removal of the C-terminal antigen region for the collagen I antibody. Additional collagen I bands at approximately 72 kDa, 55 kDa and 43 kDa in pEDS fibroblasts and supernatants indicate cleavage of a proportion of collagen I. Differences in collagen I fragmentation may be attributed to the fact that the cells come from different donors and carry different *C1R* variants, as indicated. Expression of alpha tubulin was analysed as loading control. This figure is representative of three independent experiments with similar results.

To investigate the expression of collagen I in pEDS patients, western blots from cell extracts and cell supernatant from fibroblasts were performed using a collagen I-specific antibody raised against the C-terminal globular domain of collagen I (Abcam) (heterozygous pEDS variants are c.149-150TC>AT, p.V50D; c.277G>T, p.G93C; c.902G>C, p.R301P; and c.926G>T, p.C309F, respectively). As shown in **Figure 1**, all fibroblasts expressed a collagen I chain at approximately 150 kDa in cell extracts as well as in cell supernatant. The collagen I signal in the supernatant of control 1 was very low, probably indicating very rapid processing of collagen I in this cell line. Additional smaller collagen I fragments at approx. 72 kDa and variably 55 kDa and 43 kDa were present in patient samples only. These results suggest that pre-procollagen I is cleaved intracellularly and secreted into the cell culture supernatant in pEDS fibroblasts.

### Collagen I amount is strongly reduced in the extracellular matrix of pEDS fibroblasts

**Figure 2:**
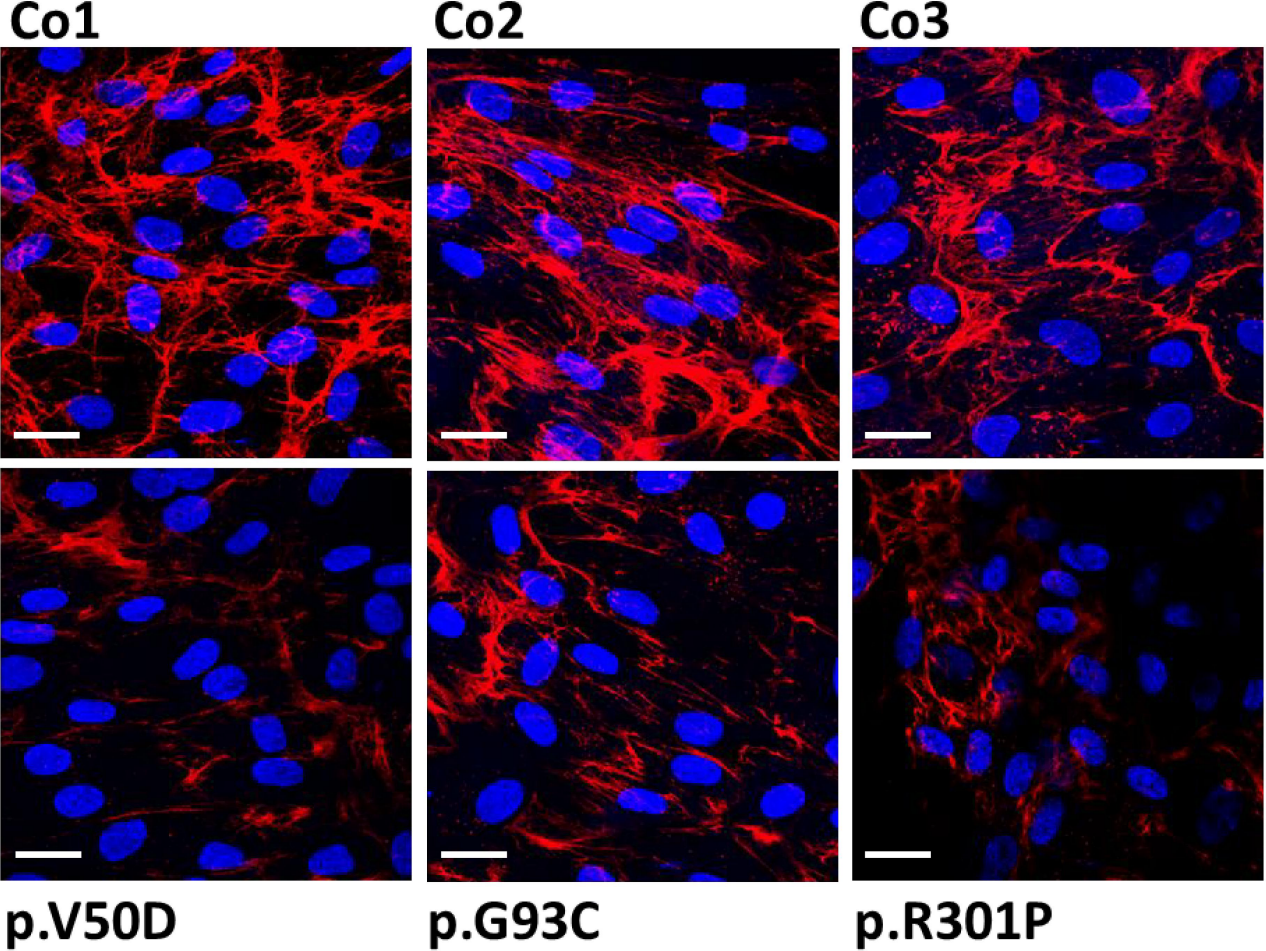
Immunofluorescence analysis of collagen I in control (Co) and pEDS fibroblasts. Control fibroblasts exhibit a high abundant collagen network with strong fiber-like staining in the extracellular space. In patient fibroblasts, the quantity of the collagen network is strongly reduced and the fiber-like staining is weaker and thinner. This is a representative figure of four independent experiments. Scale = 20 µm.

Because of the presence of collagen I fragments in the cell culture supernatant, we speculated that collagen I matrix formation may be impaired in patient fibroblasts. Thus, we performed immunofluorescence studies and confocal microscopy of control and patient fibroblasts. Cells were grown on cover slips for 7 days, then fixed and analyzed without permeabilization. In control fibroblasts extracellular collagen I staining revealed strong fibrous bundles of intense fluorescence staining predominately at the cell-cell border (**Figure 2**). In contrast, patient fibroblasts showed thin and short signals of collagen I staining. Additionally, the fluorescence signals were significantly weaker in patient cells, indicating that collagen I deposition into the extracellular matrix is strongly reduced in pEDS patients.

### Enhanced collagen I degradation and turnover in pEDS fibroblasts

**Figure 3:**
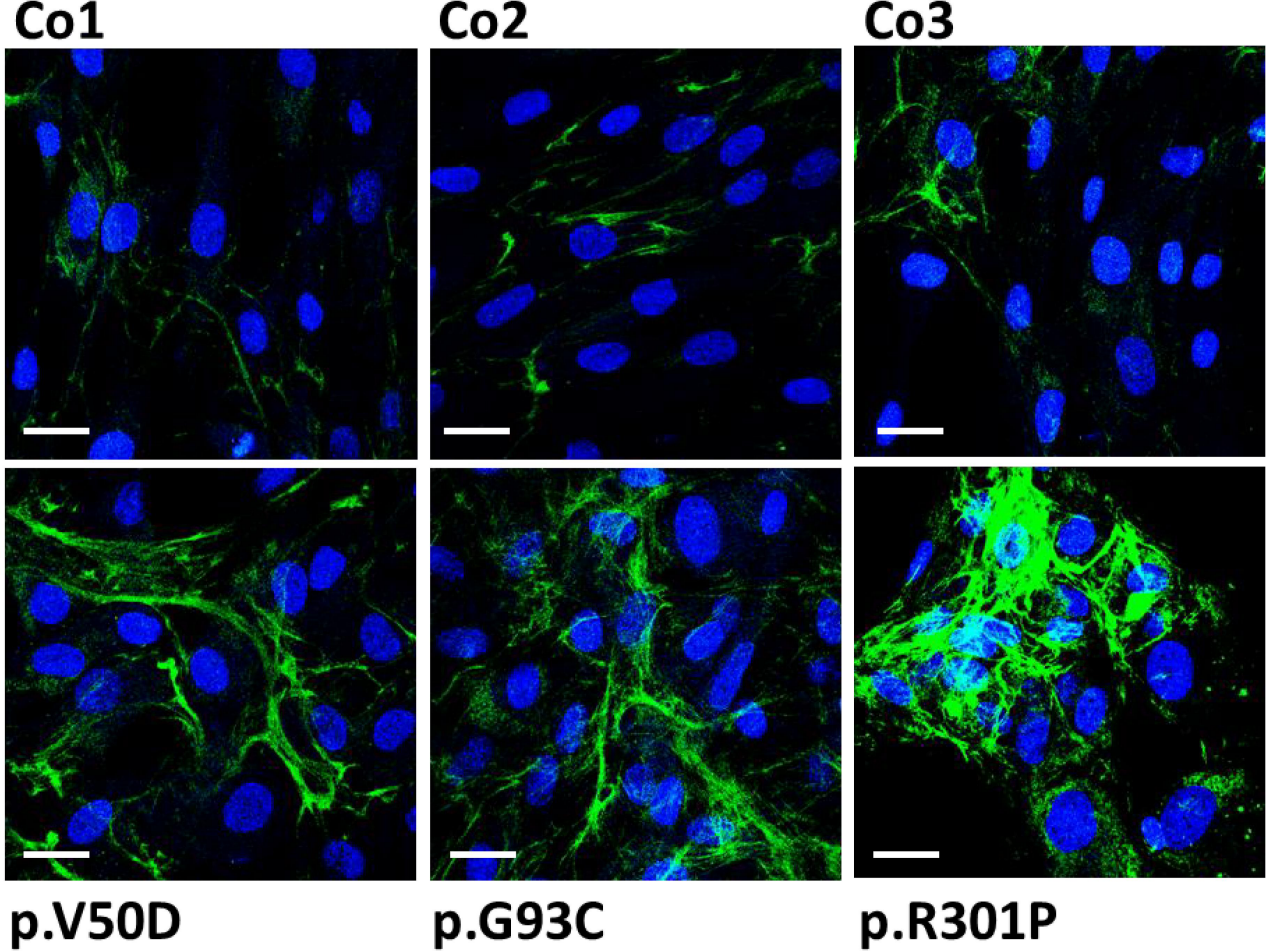
Fluorescence images showing F-CHP staining on human control (Co) and pEDS fibroblasts. Fixed cells were stained using a fluorescence-labeled collagen-hybridizing peptide (CHP) which specifically binds to denatured or fragmented collagen fibers. In controls, F-CHP signals were weak indicating slow collagen I degradation. In patient fibroblasts, F-CHP was strongly increased with thick fiber-like structures on the cell surface indicating large amounts of denatured collagen I filaments. This figure is representative of four independent experiments. Scale = 20 µm.

The potential impact of secreted collagen I fragments on collagen triple helix formation was further studied using the collagen hybridizing peptide (CHP), a unique peptide that specifically binds to unfolded collagen chains. CHP visualizes the extracellular collagen matrix turnover in tissue remodeling, or assesses collagen denaturation in disease progression. Cy3 labelled CHP was added to fixed cells, and CHP binding to denatured collagen was analyzed by confocal microscopy. In control fibroblasts, only weak signals were found predominantly in small areas on the cell surface (**Figure 3**). In contrast in patient fibroblasts, CHP hybridization resulted in strong intense bundle-like signals covering almost the whole cell surface and the cell-cell borders (**Figure 3**). These data indicate a high amount of denatured or fragmented collagen in the extracellular space of patient fibroblasts. We assume that the formation of a stable extracellular collagen matrix is severely impaired in pEDS patients.

### C1s knock down decreased collagen I degradation in fibroblasts

**Figure 4:**
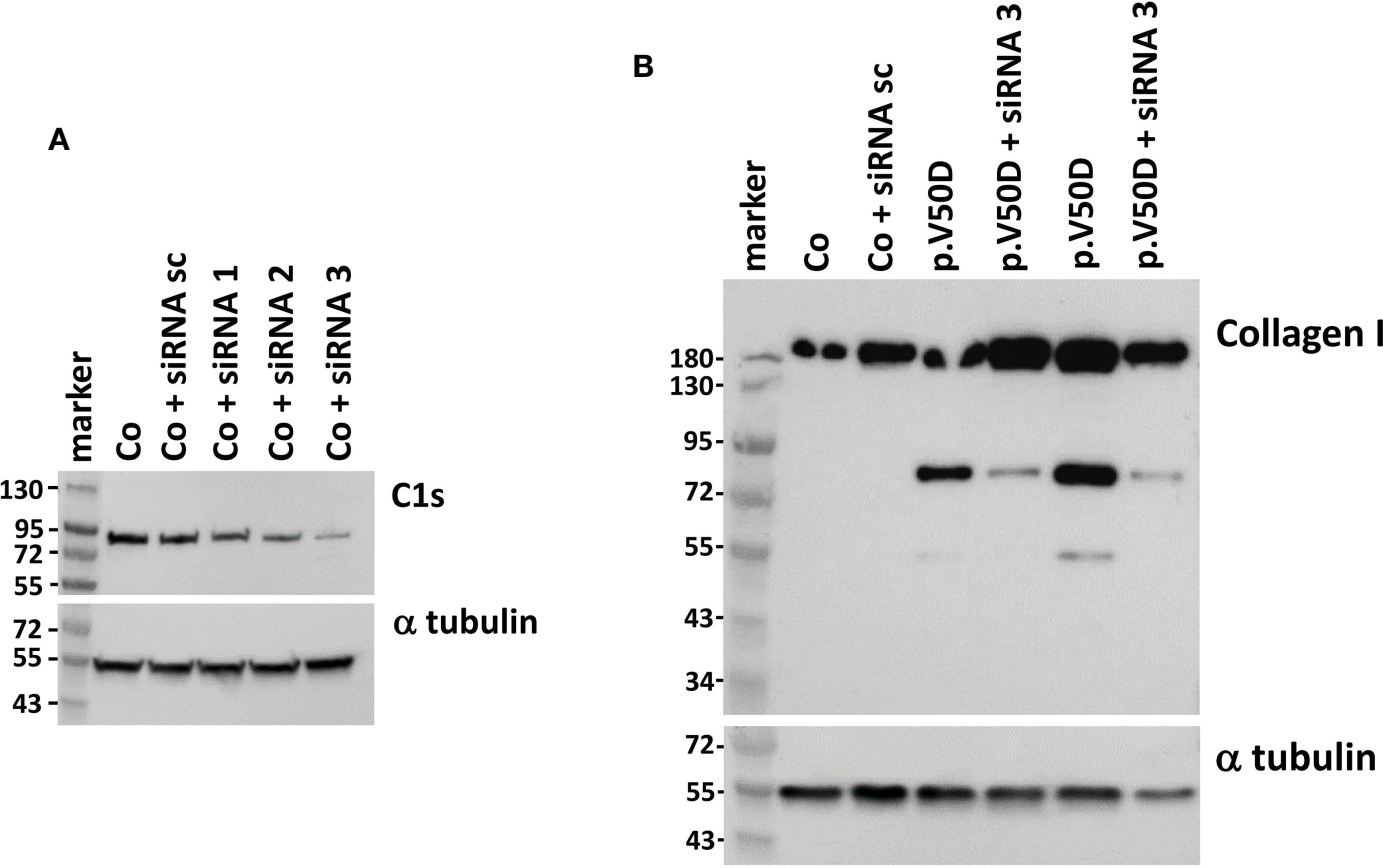
Western blot analysis of C1s and collagen I in human fibroblasts with and without *C1S* knock-down. **(A)** Transfection of the same control fibroblast with different *C1S*-directed siRNAs identifies siRNA3 as the one that causes the strongest reduction of C1s expression. **(B)** siRNA3 transfection of pEDS fibroblasts leads to marked reduction of 72 kDa and 55 kDa collagen fragments, confirming C1s as cause for collagen I digestion in patient cells. Data are representative of three independent experiments.

Yamaguchi et al. (13) showed that activated C1s (aC1s) partly cleaves collagen I and II in an *in vitro* assay. As C1s is activated in pEDS cells, we wondered if aC1s is the cause for the generation of collagen I fragments in patient cells. Therefore, we performed C1s knock down experiments to test if this influences the expression of collagen I fragments. A series of commercially available C1s specific siRNAs were tested, showing that siRNA3 strongly reduced C1s levels in human fibroblasts (**Figure 4A**). In control fibroblasts, expression of procollagen I at approximately 150kDa was unchanged after siRNA3 treatment. In contrast, treatment of patient fibroblasts with siRNA3 resulted in a marked reduction of collagen I fragments at 72 and 55 kDa, respectively, whereas collagen I at 150 kDa was unchanged (**Figure 4B**). This indicates that C1s is the cause of collagen I cleavage in pEDS fibroblasts.

### C1s degrades denatured collagen I *in vitro*

**Figure 5:**
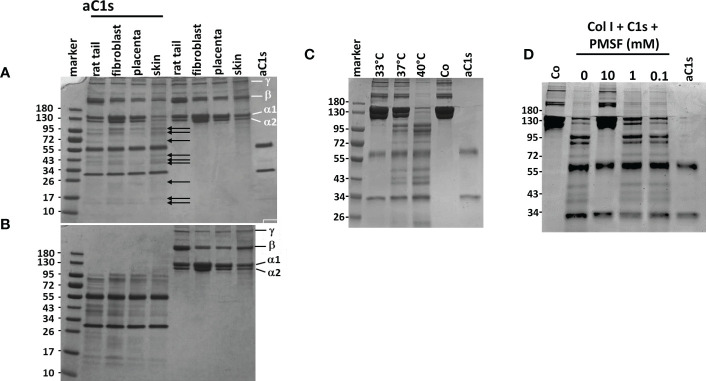
Proteolytic activity of aC1s on procollagen I **(A)** Overnight incubation of collagen I from different sources with aC1s shows partial degradation into different smaller fragments as indicated by arrows. The degradation was independent from the source of collagen I Samples incubated without aC1s remained unaffected, as shown by the α1,α2, ß and γ collagen I chains. aC1s was represented by two bands, corresponding to the N-terminal chain at approx. 65 kDa and the C-terminal chain at approx. 32 kDa. **(B)** Denaturation of collagen I prior to incubation with aC1s shows almost complete degradation of procollagen I fibers into several smaller fragments. Samples incubated without aC1s remain unchanged. **(C)** C1s-mediated degradation of collagen I is temperature-dependent, with no degradation at an incubation temperature of 33°C, limited degradation at 37°C, and almost complete degradation at 40°C. These data suggest that the collagenase activity of aC1s depends on the unfolded state of collagen I **(D)** C1s collagenase activity is abolished by addition of the serine protease inhibitor PMSF at 10mM concentration, but lower PMSF concentrations have no inhibitory effect. The data are representative of five independent experiments.

In the experiments mentioned so far, as well as in the report of Yamaguchi et al. (13), only a proportion of collagen I was found to be degraded by aC1s. We therefore examined whether this observation is explained by different types or different degrees of denaturation of collagen I. First, we incubated collagen I from different sources with aC1s as indicated in the figure legend (**Figure 5A**). The alpha, beta and gamma collagen I bands in coomassie blue stained gels were unchanged in samples incubated without aC1s. When samples were incubated with aC1s, additional collagen I fragments as indicated by arrows were found in the gel indicating that a similar proportion of collagen I is cleaved by aC1s irrespective of its origin (**Figure 5A**). Interestingly, the proportion of degraded collagen I was not changed when the incubation period was extended to 48 h or when additional activated C1s was added after 16 h (not shown). These data suggest that some collagen I chains were inaccessible to cleavage by activated C1s. We then denatured collagen I at 87°C for 10 min before incubation of samples with activated C1s at 37°C. This pretreatment led to complete aC1s-mediated degradation of the alpha, beta, and gamma collagen I chains from all sources, with all remaining bands travelling faster than the alpha band in the gel. Parallel experiments in which collagen I was incubated without aC1s showed no degradation products (**Figure 5B**).

To determine whether temperature-dependent differences in the degree of collagen I denaturation may influence aC1s-mediated collagen I cleavage, collagen I from human fibroblasts was incubated with aC1s at 33°C, 37°C, and 40°C, respectively. As shown in **Figure 5C**, collagen I was not degraded at 33°C, partly at 37°C, and completely at 40°C, indicating that the proportion of denatured collagen I determined the extent of collagen I cleavage. The results also showed that an assay temperature at 40°C is sufficient to almost cleave all collagen I chains by aC1s.

### C1s mediated collagen I degradation is blocked by serine protease inhibitors

Since C1s is a serine protease, we repeated the C1s cleavage assay in the presence of the serine protease inhibitor PMSF. As shown in **Figure 5D**, collagen I degradation was inhibited at 10 mM PMSF but not at lower PMSF concentrations. This observation indicates that inhibition of C1s protease activity can prevent collagen I degradation.

## Discussion

One of the main functions of the connective tissue is the provision of stability and protection to the organs of the body. Disorders that are associated with a generalized disturbance of these functions – the Ehlers-Danlos syndromes, EDS – are usually caused by deficient production and/or processing of extracellular matrix (ECM) components, in particular different collagens and modifying enzymes (2). Periodontal Ehlers Danlos Syndrome (pEDS) is unusual as it is caused by variants in the C1r and C1s subunits of the complement C1 complex (4). Activation of the C1 complex, typically initiated by binding to antigen-antibody complexes, triggers the classical complement cascade that is an essential process of the immune defence against pathogens (10). Consequently, uncontrolled C1r/C1s-mediated initiation of the classical complement pathway is also one of the potential pathogenic mechanisms in pEDS. Our previous studies identified extracellular presence of activated C1s (aC1s) as the common pathological feature in pEDS patient fibroblast cultures, and showed highly significant activation of complement 4 (C4) after its addition to pEDS but not control fibroblast supernatant *in vitro* (10). Animal studies have shown that periodontitis is associated with local microbiologically induced complement activation (18, 19), and therapeutic inhibition of C3 has been suggested as promising therapeutic options for periodontitis which has already entered clinical trials (20, 21).

However, complement analysis in pEDS patients does not usually show any abnormalities (4), and beyond activation of the complement components C4 and C2, C1s has been shown to act on wide range of cellular protein and regulatory pathways (12). There is no evidence that C4 is indeed the main target of activated C1s outside the C1 complex *in vivo*, and uncontrolled production of aC1s may have various complement-independent effects depending on the molecular availability of other substrates. Generalized lack of attached gingiva in the absence of periodontitis is the earliest known feature of pEDS in children (22), which seems to develop without the inflammatory phenotype expected for complement activation. Therefore, other connective tissue components that are potential substrates of the serine protease function of aC1s may be more relevant for pEDS pathogenesis, particularly if they are more abundant than C4 in the periodontal region. Prime candidate for this effect is collagen 1.

A causative link between EDS and collagen 1 is well recognized. Apart from the role of pathogenic type I collagen variants for the development of osteogenesis imperfecta (OI) or brittle bone disease, specific variants in *COL1A1* or *COL1A2* have been identified as causes of different EDS types such as arthrochalasia EDS, cardiac-valvular EDS, as well as rare forms of vascular EDS and classic EDS and OI-EDS overlap syndromes (3, 23–27). Work by Yamaguchi and coworkers (13) showed that aC1s cleaved type I collagen, the main component of the ECM fibres. Collagen I is a fibrillary molecule composed of two α1 chains and one α2 chain ([α1]2[α2]1) encoded by *COLIA1* and *COLIA2* genes, respectively (28). The collagen I proteins are translated into the ER as pre-propeptides. Processing within the ER comprises removal of the N-terminal signal sequence, vitamin C-dependent hydroxylation of lysine and proline residues, glycosylation of hydroxylysine, and finally C-to-N-terminal assembly into heterotrimeric procollagen. The newly formed trimer is moved to the Golgi apparatus for final modifications, assembled into specialized secretory vesicles, and released into the extracellular space. Outside the cells, procollagen is enzymatically cropped by removing N-propeptide (N-pp) and C-propeptide (C-pp) to form tropocollagens, which are crosslinked by lysyl oxidyase-mediated covalent bonding to form collagen fibres (for reviews see (28) (29)).

Our experiments showed that in pEDS fibroblasts, a considerable portion of intracellular procollagen I is cleaved by aC1s. The collagenolytic activity of aC1s is clearly temperature-sensitive as shown in *in vitro*: at 33°C no collagen I was cleaved whereas at 40°C almost all collagen I was degraded. aC1s-mediated collagen degradation thus depends on the degree of unfolded collagen I fibrils (**Figure 5**). It is tempting to suggest that a similar process may also take place in the extracellular space: collagen I is thermally unstable at 37°C, with local melting and refolding required for adequate fibrillogenesis. By using differential scanning calorimetry, Leikina and colleagues showed that the preferential structure of collagen I is a random coil rather than a triple helix at body temperature (30). They also point out that even a small decrease in stability has a dramatic effect on collagen unfolding at body temperature.

Collagen breakdown is an integral element of tissue homeostasis and repair, involving digestion of collagen into small fragments and removal from the extracellular space (31). Our data suggest that increased collagen I cleavage by aC1s shifts collagen turnover toward higher collagen I resorption, resulting in thinner and shorter collagen I fibers as found in pEDS fibroblasts (**Figure 2**). This effect seems to be more pronounced at higher temperatures as found in inflamed tissues. Breakdown of collagen and potentially other extracellular matrix proteins may thus represent a relevant additional function of C1s in immune defense beyond activation of the classical complement pathway as it may assist in loosening the extracellular matrix to enhance access to inflammatory cells.

The exact mechanism of the interaction between aC1s and collagen I at the cleavage sites remains to be clarified. Interestingly, a calcium dependent interaction of collagen-lysine and C1s CUB1 module was shown previously, and mutations in the calcium-binding site strongly alter the binding capacity of C1s to the collagen-like stalk of C1q (7, 32). Structural analysis of the interaction between the procollagen C-proteinase enhancer 1 and the procollagen triple helix showed that the CUB1-CUB2 domains from the proteinase enhancer bind to two different chains of procollagen III trimer, unravelling the procollagen molecule in the region of the proteolytic cleavage site and thereby facilitating the action of the associated proteinase (33). Given the high structural and functional conservation of different CUB domains (7, 34), a similar binding mechanism is not unlikely for C1s binding to procollagen.

In conclusion, our study supports the suggestion that a direct proteolytic effect of activated C1s on collagen and possibly other connective tissue components is the primary pathogenic mechanism in pEDS. Prominent manifestation in the periodontal region is likely due to the overexpression of complement 1 proteins at a position of high exposure to exogenous microorganisms. Involvement of other organ systems such as the skin, joints, blood vessels, and brain, could be linked to variable low-grade C1r/C1s expression. It is interesting to note that in an older study, purified C1s inhibited collagen-induced platelet aggregation (35), which would be a possible explanation for easy bruising and the pretibial discoloration observed in pEDS. Our study is the first to demonstrate overproduction of an ECM protease as pathomechanism for a specific EDS type, and confirms the classification of pEDS as a primary connective tissue disorder. Protease overactivation may also play a role in other related conditions such as hypermobile EDS, the molecular cause of which has so far remained elusive. Further studies should examine whether blockage of protease activity – possibly by local administration of an appropriate inhibitor – may be an effective therapeutic approach for pEDS and possibly other disorders.

## Data availability statement

The raw data supporting the conclusions of this article will be made available by the authors, without undue reservation.

## Ethics statement

The analyses were approved by the Clinical Research Ethics Board of the Medical University Innsbruck (UN4501 and 1074/2017). The patients/participants provided their written informed consent to participate in this study.

## Author contributions

JP, PT, NT, CG, and LK performed experiments and analysis. AA, IK-S, HS, and JZ wrote and edited the manuscript. All authors contributed to the article and approved the submitted version.
